# Should All Patients With HR-Positive HER2-Negative Metastatic Breast Cancer Receive CDK 4/6 Inhibitor As First-Line Based Therapy? A Network Meta-Analysis of Data from the PALOMA 2, MONALEESA 2, MONALEESA 7, MONARCH 3, FALCON, SWOG and FACT Trials

**DOI:** 10.3390/cancers11111661

**Published:** 2019-10-26

**Authors:** Valentina Rossi, Paola Berchialla, Diana Giannarelli, Cecilia Nisticò, Gianluigi Ferretti, Simona Gasparro, Michelangelo Russillo, Giovanna Catania, Leonardo Vigna, Rossella Letizia Mancusi, Emilio Bria, Filippo Montemurro, Francesco Cognetti, Alessandra Fabi

**Affiliations:** 1 Breast Unit, S. Camillo-Forlanini Hospital of Rome, 00152 Rome, Italy; VRossi@scamilloforlanini.rm.it (V.R.); LVigna@scamilloforlanini.rm.it (L.V.); 2 Department of Clinical and Biological Sciences, University of Turin, 10124 Turin, Italy; paola.berchialla@unito.it; 3 Department of Medical Statistics, IRCCS Regina Elena National Cancer Institute, 00128 Rome, Italy; Diana.giannarelli@ifo.gov.it; 4 Division of Medical Oncology1, IRCCS Regina Elena National Cancer Institute, 00128 Rome, Italy; cecilia.nistico@ifo.gov.it (C.N.); gianluigi.ferretti@alice.it (G.F.); simona.gasparro@ifo.gov.it (S.G.); michelangelo.russillo@ifo.gov.it (M.R.); giovanna.catania@ifo.gov.it (G.C.); 5 Department of Statistical Sciences, University Tor Vergata of Rome, 00133 Rome, Italy; mncrsl01@uniroma2.it; 6 Comprehensive Cancer Center, Fondazione Policlinico Universitario Agostino Gemelli IRCCS, 00168 Rome, Italy; emiliobria@yahoo.it; 7 Department of Medical Oncology, Università Cattolica del Sacro Cuore, 00168 Rome, Italy; 8 Direzione Day Hospital Oncologico Multidisciplinare, Istituto di Candiolo, FPO-IRCCS, 10060 Candiolo, Italy; Filippo.montemurro@ircc.it; 9 Università La Sapienza, 00185 Rome, Italy; francesco.cognetti@ifo.gov.it

**Keywords:** palbociclib, ribociclib, abemaciclib, fulvestrant, aromatase inhibitors, metastatic breast cancer

## Abstract

*Background*: We aim to understand whether all patients with hormonal receptor (HR)-positive (+)/human epidermal growth factor receptor-2 (HER2)-negative (−) metastatic breast cancer (MBC) should receive cyclin D-dependent kinase (CDK) 4/6 inhibitor-based therapy as a first-line approach. Methods: A network meta-analysis (NMA) using the Bayesian hierarchical arm-based model, which provides the estimates for various effect sizes, were computed. Results: First-line treatment options in HR+/HER2− MBC, including CDK 4/6 inhibitors combined with aromatase inhibitors (AIs) or fulvestrant (F), showed a significantly longer progression-free survival (PFS) in comparison with AI monotherapy, with a total of 26% progression risk reduction. In the indirect comparison across the three classes of CDK 4/6 inhibitors and F endocrine-based therapies, the first strategy resulted in longer PFS, regardless of specific CDK 4/6 inhibitor (HR: 0.68; 95% CrI: 0.53–0.87 for palbociclib + AI, HR: 0.65; 95% CrI: 0.53–0.79 for ribociclib + AI, HR: 0.63; 95% CrI: 0.47–0.86 for abemaciclib + AI) and patient’s characteristics. Longer PFS was also found in patients with bone-only and soft tissues limited disease treated with CDK 4/6 inhibitors. Conclusions: CDK 4/6 inhibitors have similar efficacy when associated with an AI in the first-line treatment of HR+ MBC, and are superior to either F or AI monotherapy, regardless of any other patients or tumor characteristics.

## 1. Introduction

Hormone receptor (HR)-positive (+) and human epidermal growth factor receptor type (HER2)-negative (−) metastatic breast cancer (MBC) represents the most common invasive cancer subtype in women [1]. Almost two-thirds of women with newly diagnosed MBC have HR+ tumors, and approximately 25% of women with an early HR+ breast cancer diagnosis eventually relapse after adjuvant treatments [2]. 

As the role of estrogens in etiology and breast cancer progression is well-established, the modification of estrogen activity has represented the treatment of choice in women with HR+ MBC for several years, particularly for those with slowly progressive disease and limited tumor-related symptoms [3]. Selective estrogen receptor modulators (such as tamoxifen) [4], selective estrogen receptor down-regulators (SERDs, like fulvestrant [F]) [5,6] aromatase inhibitors (AIs; such asletrozole [7], anastrazole [8] or exemestane [9]) are the mainstay of anticancer endocrine therapy (ET) [10]. The recent addition of CDK 4/6 inhibitors to standard ET has further improved outcomes in both first- and later-line therapy settings. CDK 4/6 inhibitors act by inactivating the complex CDK-D-type cyclins (CCND), leading to an increase in the retinoblastoma protein (pRb), which negatively regulates E2F transcriptional factors, eventually resulting in the inhibition of cell cycle progression and apoptosis of tumor cells [11]. 

In the first-line setting, CDK 4/6 inhibitors, which mechanistically work in different ways through estrogen receptor interference, have been studied in combination with AIs in the PALOMA-2 [12], MONALEESA-2 [13], and MONARCH-3 studies in the context of postmenopausal women [14], as well as in premenopausal women, in combination with either tamoxifen or an AI in the MONALEESA-7 study [15]. In particular, palbociclib, ribociclib, and abemaciclib, the three available CDK 4/6 inhibitors, in combination with standard ET, showed progression-free survival (PFS) improvement in phase III trials, and have been approved for use in first and in later lines of therapy in women with HR+HER2− MBC, regardless of menopausal status, age, endocrine sensitivity and type of metastasis [12,16]. Interestingly, although the mechanism of action of these CDK 4/6 inhibitors is similar, they also present some differences. In fact, while palbociclib and ribociclib have a similar chemical structure, abemaciclib is 14-times more potent against CDK 4 compared with CDK 6 and presents a higher selectivity for the complex CDK 4/cyclin D1 [11].

Despite the progress made in efficacy, combination therapy also resulted in increased toxicities, costs, and tighter clinical monitoring for patients [17]. In addition, the improvement in PFS has not yet translated into an increase in overall survival (OS) for all studies focusing on first-line settings [18,19,20].

Therefore, how and when to incorporate CDK 4/6 inhibitors in the complex management of HR+ HER2− MBC remains one of the main unmet clinical need in this setting [21].

Indeed, single-agent ET yielded a median PFS ranging from 14 to 16 months in the control arms of the first-line trials with CDK 4/6 inhibitors. Thus, the fact that for some patients, the addition of CDK 4/6 inhibitors might be avoided is a debated clinical topic. Moreover, according to the Fulvestrant and Anastrozole Compared in Hormonal Therapy Naive Advanced Breast Cancer (FALCON) trial, comparing F with anastrozole in the same setting, the single-agent F therapy may be a further reasonable option for HR+/HER2− MBC patients who are ET-naïve, especially those with the non-visceral disease. Indeed, the median PFS of 16.6 months reported in the F arm compared with 13.3 months in the anastrozole arm, which was observed in the context of the general population, was even higher in patients without visceral disease (22.3 versus 13.6 months; hazard ratio [HR]: 0.59; 95% CI: 0.42–0.84) [5].

The ongoing studies, which are evaluating predictive markers of endocrine resistance or sensitivity, will probably provide enough evidence for helping in the clinical decision-making and for a better definition of the optimal ET-based strategy for this specific set of luminal breast cancer [22,23,24].

In the meantime, in our current daily clinical practice, also considering minor or uncertain differences in efficacy between the available CDK 4/6 inhibitors, the choice of first-line ET strategy is essentially based on one of the three CDK 4/6 inhibitors according to their specific toxicity profile and patient comorbidities or preferences.

Considering the lack of formal and reliable comparisons between the three CDK 4/6 inhibitors, in addition to the lower toxicity profile of F, the aim of this systematic review and meta-analysis is the indirect comparison between the combination strategy, including CDK 4/6 inhibitors plus AI [12,13,14,15], and F-based therapies for the first-line treatment of HR+/HER2− MBC [5,25,26,27].

## 2. Materials and Methods

### 2.1. Search Strategy and Study Selection

We followed the PRISMA statement for reporting systematic reviews and meta-analysis. Two authors independently examined the abstracts retrieved by a search strategy in electronic databases (MEDLINE, EMBASE, and The Cochrane Central Register of Controlled Trials) from November 2011 to June 2019. We used the following search string ((metastatic breast cancer) AND (CDK 4/6 inhibitor OR endocrine therapy OR aromatase inhibitor OR letrozole OR anastrozole OR exemestane OR tamoxifen OR F OR palbociclib OR everolimus OR ribociclib OR abemaciclib). The research was conducted on 5 June 2019. Proceedings of the American Society of Clinical Oncology (ASCO) Annual Meeting, San Antonio Breast Cancer Annual Symposium, and the European Society of Medical Oncology Annual Meeting were also queried from November 2011 to June 2019 for relevant abstracts. In cases where a report of the same trial was obtained, the most recent results were included (corresponding to longer follow-up). Then, the authors examined full-text articles of potentially eligible studies according to the eligibility criteria. Disagreements on the inclusion of selected trials were resolved in discussions with another author. This article does not contain any studies with human participants or animals performed by any of the authors. It was unnecessary, given the study does not contain any studies with human participants or animals performed by any of the authors.

### 2.2. Eligibility Criteria

We decided only to include phase III randomized controlled trials (RCTs) that reported the comparison of CDK 4/6 inhibitors plus ET or F plus or less ET versus ET treatment alone as first-line treatment in HR+/HER2− MBC. We also excluded trials with incomplete data or different control arm.

### 2.3. Outcomes

The primary outcome was PFS, calculated from the date of randomization to the date of progression (defined by the Response Evaluation Criteria in Solid Tumors “RECIST” 1.1 criteria or death). The secondary outcomes were: (1) objective response rate (ORR): defined as the percentage of patients with complete or partial response as per RECIST 1.1 criteria (as assessed in all randomly assigned patients); (2) clinical benefit (CB): defined as a confirmed complete or partial response or stable disease lasting 24 weeks or more; (3) OS: defined as the time from randomization to death from any causes. Subgroup meta-regression analysis was also conducted for PFS indirect comparison according to age, Eastern Cooperative Oncology Group (ECOG) performance status, ethnicity, prior chemotherapy or ET exposure, measurable disease at the time of metastasis occurrence, visceral or bone-only disease, time from the initial diagnosis of breast cancer to metastasis onset. 

### 2.4. Data Collection and Statistical Analysis

A network meta-analysis (NMA) was carried out utilizing the method in the study by Valkenhoef et al. [28], which performed NMA using the Bayesian hierarchical arm-based model and provides estimates for various effect sizes. For PFS, HR and 95% credible interval (CrI) were reported. In addition, ORR and CB rates were reported, and the results were expressed as odds ratios (OR) with their 95% CrI. The NMA plot, in which treatments directly compared were connected with straight line was generated. NMAs on patients’ subgroups were also performed. All analyses were performed using R Statistical Software version 3.4.3 along with the gemtc package, which uses Markov chain Monte Carlo (MCMC) techniques through Just Another Gibbs Sampler (JAGS) [29].

## 3. Results

### 3.1. Study Selection

**Figure 1:**
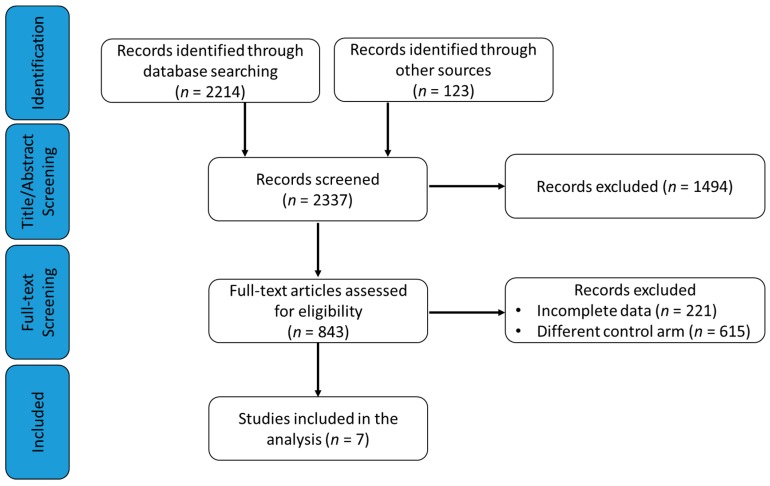
The Preferred Reporting Items for Systematic Reviews and Meta-Analyses (PRISMA) flow chart summarizing the process for the identification of the eligible studies.

Through the search strategy, we identified four phase III trials comparing 1441 patients treated with CDK 4/6 inhibitors (palbociclib, ribociclibor, abemaciclib) in combination with an AI (1106 patients) [12,13,14], an AI plus ovarian function suppression (OFS; 248 premenopausal patients), or tamoxifen plus OFS (87 premenopausal patients) [15]. Three other phase III randomized controlled trials (RCTs) compared 837 patients treated with F alone (230 patients) or in combination with AI (607 patients) versus a total of 1891 patients treated with AI alone (letrozole 2.5 mg daily or anastrozole 1 mg per day on a continuous schedule), tamoxifen plus OFS (90 premenopausal patients) or AI plus OFS (247 premenopausal) [5,25,27]. The Preferred Reporting Items for Systematic Reviews and Meta-Analyses (PRISMA) flow diagram for study inclusion is shown in Figure 1.

### 3.2. Description of Studies and Patients

**Figure 2:**
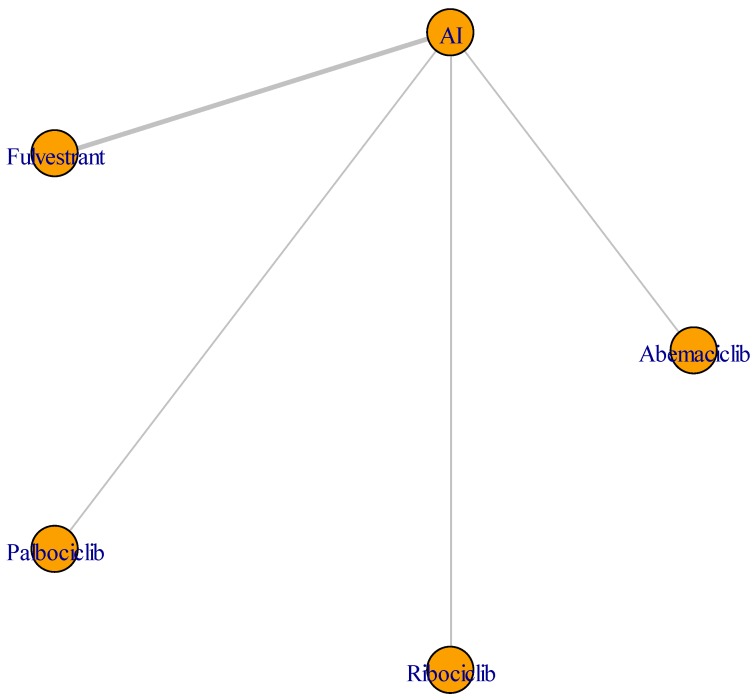
The network meta-analysis design: Network plot of the network meta-analysis. AI: Aromatase inhibitor.

Table 1 summarizes the main characteristics and outcomes of each trial. Palbociclib and ribociclib were tested in combination with letrozole 2.5 mg/day in PALOMA-2 and MONALEESA-2, respectively. Abemaciclib was used in combination with anastrozole 1 mg/day (19.9%) or letrozole 2.5 mg/day (79.1%) as per the physician’s choice in the MONARCH-3 trial. Furthermore, ribociclib was also studied in premenopausal patients in the context of the MONALEESA-7 trial. Specifically, in this study, ribociclib was combined with tamoxifen plus goserelin (26%) or with letrozole 2.5 mg/day or anastrozole 1 mg/day plus goserelin (74%). Eventually, F alone or in combination with anastrozole 1 mg/day was compared with the AI in the FALCON, Southwest Oncology Group (SWOG), and Fulvestrant and Anastrozole Combination Therapy (FACT) trials. The primary outcome was PFS in all trials (Table 1); secondary outcomes (ORR, CB rate, OS) in all trials were also reported in Table 1, while the NMA core design is shown in Figure 2. The network plot in Figure 2 offers a visual representation of the evidence. Nodes represent treatments, and edges represent the available direct comparisons—that is, they connect treatments that are directly compared in studies. 

### 3.3. Outcomes

#### 3.3.1. Progression-Free Survival

**Figure 3:**
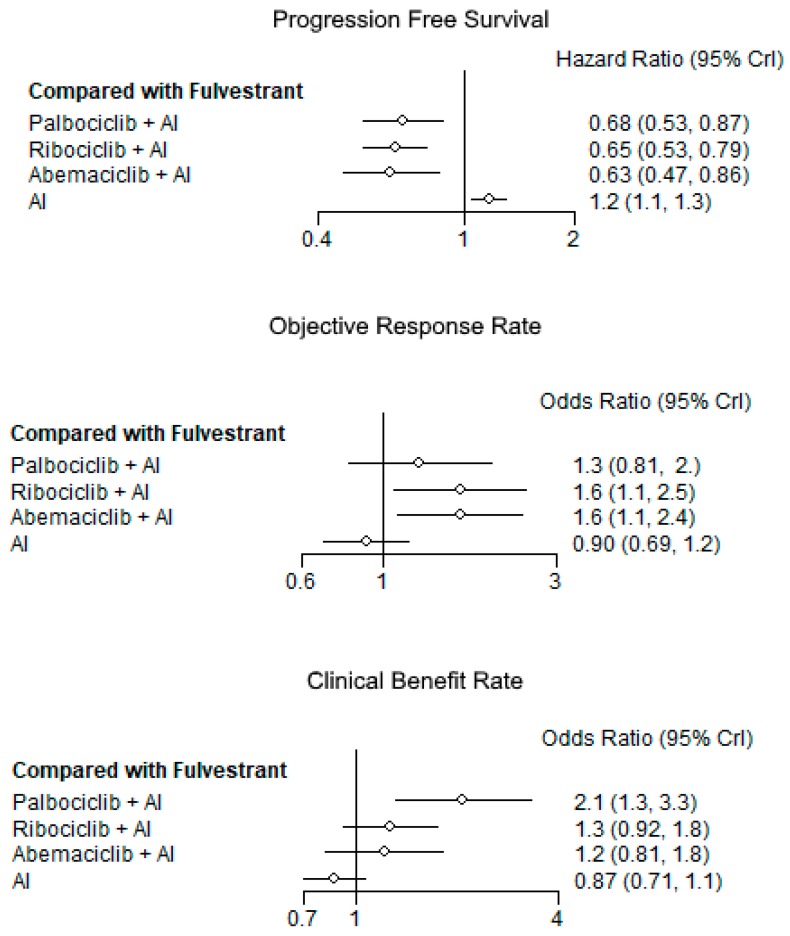
Forest plots with direct comparisons against fulvestrant for progression-free survival, objective response rate, and clinical benefit rate. AI: Aromatase inhibitor.

As shown in the indirect comparison between CDK 4/6 inhibitors versus F-based therapies, the first strategy resulted in longer PFS, regardless of the specific CDK 4/6 inhibitor (HR: 0.68; 95% CrI: 0.53–0.87 for palbociclib + AI, HR: 0.65; 95% CrI: 0.53–0.79 for ribociclib + AI, HR: 0.63; 95% CrI: 0.47–0.86 for abemaciclib + AI) (Figure 3; top)

#### 3.3.2. Objective Response

The CDK 4/6 inhibitors combination strategies resulted in higher RR in indirect comparison with F (OR:1.3; 95% CrI: 0.81–2.0 from palbociclib + AI versus AI: OR:1.6; 95% CrI: 1.1–2.5 from ribociclib + AI versus AI: OR:1.6; 95% CrI: 1.1–2.4 from abemaciclib + AI versus AI). (Figure 3; middle)

#### 3.3.3. Clinical Benefit

The CDK 4/6 inhibitor combination strategies resulted in higher CB in indirect comparison with F (OR: 2.1; 95% CrI: 1.3–3.3 from palbociclib + AI versus AI: OR: 1.3; 95% CrI: 0.92–1.8 from ribociclib + AI versus AI: OR:1.2; 95% CrI: 0.81–1.8 from abemaciclib + AI versus AI). (Figure 3; bottom).

#### 3.3.4. Overall Survival

For overall survival (OS), no indirect comparison by NMA was performed because not all the studies data were completely mature. Indeed, data on OS have been recently reported in the MONALEESE-7 study where the estimated OS at 42 months was 70.2% (95% CI 63.5–76.0) in the CDK 4/6 inhibitor arm versus 46% (95% CI: 32–58.9) in the ET alone arm [18,19] In addition, also the SWOG trial has recently shown the median OS of 49.8 months in 71% of the patients receiving F combination strategy vs. 42 months in 76% of the patients receiving ET monotherapy alone [23].

#### 3.3.5. Safety Profile

The main adverse effects registered in each trial are reported in Table 2.

### 3.4. Subgroup Analyses

Table 3 summarizes the main patient’s characteristic according to subgroups analysis in each trial.

**Figure 4:**
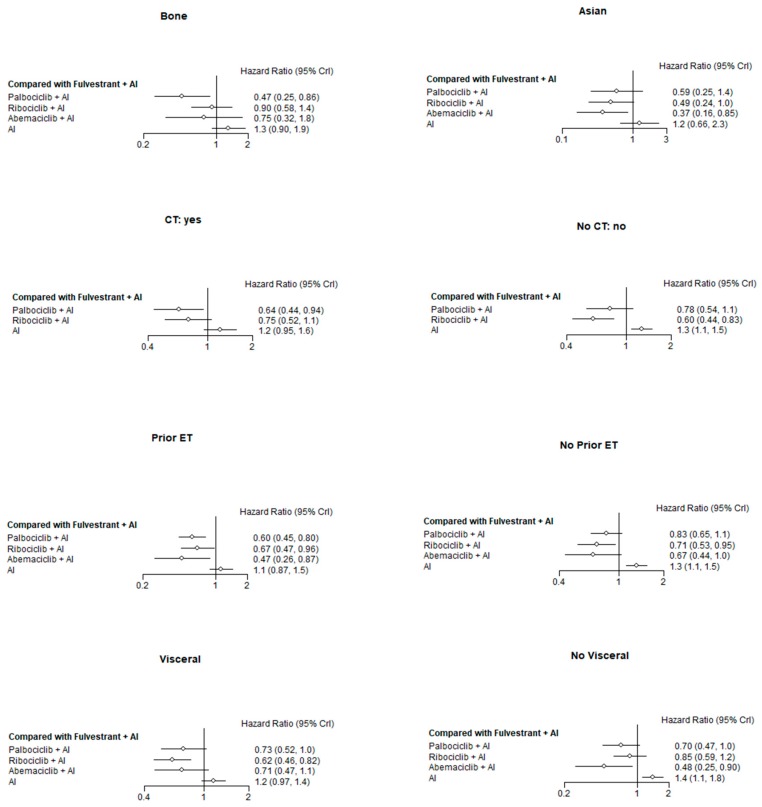
Forest plot on treatment effect for progression-free survival by subgroups in the indirect comparison between CDK 4/6 inhibitors, aromatase inhibitors, and fulvestrant. AI: Aromatase inhibitor; CT: Chemotherapy; ET: Endocrine therapy.

Subgroup NMA among the three classes of CDK 4/6 inhibitors and F is reported in Figure 4.

For PFS analysis of the seven selected phase III RCTs [5,12,13,14,15,25,27] according to prespecified subgroups, a total of 2278 patients were in the CDK 4/6 inhibitors or fulvestrant arm and a total of 1891 patients were in ET arm alone. Among them, 335 patients in the CDK 4/6 inhibitors and 337 patients in ET arms alone were premenopausal. The indirect comparison between CDK 4/6 inhibitors combination strategies and F-based therapies showed quite consistent PFS improvements in favor of CDK 4/6 inhibitors in all subgroups. With reference to the most important NMA aim, compared with F-based therapy, the CDK 4/6 inhibitor combination strategy was associated with PFS improvement also in patients with disease limited to the bone or in non-visceral sites. Although no statistically significant difference emerged among the three classes of inhibitors in indirect comparison, NMA results also suggested a different potential tropism among them, which should be further investigated in prospective clinical trials.

## 4. Discussion

HR+/HER2− BC is the most common subtype of this disease, representing approximately 60–70% of all breast tumors [1]. For many years, the sequential use of ET was the preferred approach in HR+ MBC patients due to its effectiveness and favorable toxicity profile [3,30,31]. The recent introduction of new combinations of ET plus CDK 4/6 or phosphatidylinositol 3-kinases (PI3K) inhibitors led to further clinical improvement in HR+ MBC patients [30,31]. Regarding the first-line setting, several randomized phase III clinical trials clearly demonstrated that the three highly selective CDK 4/6 inhibitors (palbociclib, ribociclib, abemaciclib) significantly improve ORR and CB and prolong PFS when combined with an AI or tamoxifen, both in pre- and post-menopausal women [12,13,14,15].

Although clinical outcomes are very similar for the three CDK 4/6 inhibitors and are not influenced by classic clinical and pathological factors, several differences were recognized concerning their toxicity profile and mechanism of action. In the patients who received the combination CDK 4/6 inhibitor plus ET, a higher incidence of hematologic adverse events occurred compared to control groups. The most common hematologic adverse events with grade 3 or 4 were neutropenia (43% vs. 1%), leucopenia (20% vs. 0.4%), anemia (5% vs. 1%), and thrombocytopenia (2% vs. 0.1%) [32]. Despite the high incidence of neutropenia reported in the RCTs, a higher incidence of febrile neutropenia was not recorded, which is evident just in 1.3% of patients. Unlike palbociclib and ribociclib, abemaciclib caused low-grade diarrhea and transaminase elevation, readily managed with conventional medications or dose reduction [17]. On the other hand, data suggest that abemaciclib has distinct single-agent activity at the molecular level, which could reflect its unique effects and toxicity profile [33]. For example, abemaciclib, but not ribociclib or pabociclib, is a potent inhibitor of kinases other than CDK 4/6, including CDK1/cyclin B, which appears to cause arrest in the G2 phase of the cell cycle, and CDK2/cyclin E/A, which is implicated in resistance to palbociclib. Whereas ribociclib and palbociclib induce cytostasis, and cells adapt to these drugs within 2–3 days of exposure, abemaciclib induces cell death and durably blocks cell proliferation. Abemaciclib is active even in pRB-deficient cells in which CDK 4/6 inhibition by palbociclib or ribociclib is completely ineffective [34]. In luminal tumors, some useful biomarkers are being studied to identify the best target treatment combined with anti-hormonal therapy and which can then determine the ideal choice for activity rather than toxicity. Recently, the *PI3K* mutation presented in progressive luminal tumors from the first-line treatment with AI showed a significant benefit in PFS when associated with F [35].

Against this background, knowing whether these three agents can be interchangeable remains an urgent unmet clinical need [21,32,36,37]. Thus, a better understanding of molecular differences is a relevant challenge since it could be informative for their right use in the clinical setting. Additionally, since there are no comparative data between CDK 4/6 inhibitors and F, the hormonal agent approved in endocrine-naïve patients with MBC for first-line setting, the identification of the most suitable HR+ MBC patients who can benefit most from the less toxic F-based-therapy is still a significant challenge [5,21]. Herein, we performed a meta-analysis, including only data from phase III RCTs available concerning the same clinical scenario. This approach allows the synthesis of a large body of evidence while retaining the benefits of randomization within each trial. Hence, we use this indirect comparison method to investigate whether F-based ET could still play a role in some specific subgroup of patients with HR+ MBC in the first-line setting

In this meta-analysis, we found that CDK 4/6 inhibitors produced significant improvement in ORR, CB, and PFS in all patients with HR+ MBC in comparison with F-based therapies. Furthermore, these results were independent of age, race, performance status, disease site, prior chemotherapy, prior ET, disease-free interval after adjuvant treatment, menopausal status, type of CDK 4/6 inhibitor or expression of the progesterone receptor. Interestingly, significant PFS improvement in favor of CDK 4/6 inhibitors was observed even in patients with bone-only disease and in non-visceral disease. Due to the lack of convincing efficacy criteria to prefer one or the other CDK 4/6 inhibitor, the choice should rely on the toxicity profile. It must be remembered, however, that no direct comparison has ever been performed between the three CDK 4/6 inhibitors to allow an appropriate selection in the clinic.

Our meta-analysis has several limitations suggesting caution in interpreting the results. First, it is not based on individual patient data but was conducted considering the HR and 95% CIs of each study extracted. Moreover, for some subgroups, the HRs were not reported in all studies, particularly in F-based trials. In addition, the number of trials was relatively limited, preventing formal comparisons among all treatment strategies for each group. Second, and this is a well-known caveat of studies with F, not all the included studies with this compound used the now considered standard dose (500 mg monthly plus an additional dose of 500 mg for 15 days during the first month) [25,27]. The CONFIRM trial has revealed a superior activity of 500 mg when compared with 250 mg of F in terms of PFS and OS [6]. Therefore, the non-standard F dose used in some trials may partially bias our findings. Third, while the four RCTs [12,13,14,15] including CDK 4/6 inhibitors are rather homogenous in terms of inclusions criteria and patient characteristics, the greater heterogeneity observed in F-based studies [5,25,27] inevitably affected the pooled meta-analyses results. For instance, the line of therapy, metastatic sites, tumor burden, prior anti-estrogen drugs, and anti-estrogen treatment sequence, previous endocrine therapies sensitivity were also heterogeneous. Notably, F was more effective in endocrine-naïve patients without the visceral disease [5]. As demonstrated in Table 1, the FALCON trial exclusively included those patients who had never been exposed to ET, both in the metastatic and early settings. By contrast, a percentage ranging from 40 to 65% of patients included in the remaining trials were previously exposed to ET in the adjuvant or neoadjuvant settings. In addition, F efficacy in “bone-only” limited disease was not established in all studies [27]. Finally, for peri and premenopausal patients, we cannot rule out the possibility that the administered luteinizing hormone-releasing hormone analog (LHRHa) may affect the final estimation, as LHRHa itself was reported as an effective endocrine approach in breast cancer. Moreover, the limited number of peri or premenopausal patients in F-based studies makes a comparison between F and CDK 4/6 inhibitor ET strategies unreliable in this setting of patients. Specifically, while the FALCON and SWOG trials were enrolled merely post-menopausal patients, in the FACT trial, premenopausal patients were also included and were treated with GnRHa. Unfortunately, they only represented 3.1% of the experimental arm. Although previous reviews and meta-analyses of treatments in MBC patients have been conducted, many of these studies focus on population different from the current study. For example, Ayyagari et al. explored the safety and efficacy of only two CDK 4/6 inhibitors (palbociclib and ribociclib) in postmenopausal women after progression on a non-steroidal AI [35]. Ding et al. also conducted a similar NMA, focusing on the results of all three CDK4/6 inhibitors in phase II and III trials both in the first and second lines; however, the analysis did not include a direct comparison with F in first-line treatment [32]. Finally, El Rassy et al. investigated which CDK4/6 inhibitor was more effective in patients with luminal breast cancer, but their study was limited by the small sample size and lack of studies on F (1 included) [36]. Therefore, our study specifically focused on the results of phase III studies in the first-line setting and comprised an indirect comparison with first-line F. In addition, the present meta-analysis includes recently published data that were not included in previous reviews, in particular, the results of MONALEESA-7 on premenopausal women [32,36,37]. Despite the differences in study design and specific limitations, the results of our and previous NMAs are consistent in reporting the improved clinical outcomes obtained with CDK4/6 inhibitors compared with monotherapy [32,36,37].

## 5. Conclusions

In conclusion, the results of this meta-analysis confirm that CDK 4/6 inhibitors have similar efficacy when associated with an AI in the first-line treatment of HR+ MBC, and are superior to either F or AI monotherapy, regardless of any other patients or tumor characteristics. Though all CDK 4/6 inhibitors resulted associated with similar outcomes, the differences in toxicity profile, drug interactions, and patient preferences seem to be the main factors to be considered in the clinical decision-making process. Interestingly, CDK 4/6 inhibitors with AI resulted in more effective than F-based therapies even in patients with the bone-limited disease and, what is more, in patients without visceral disease involvement. Hence, the use of F in the first-line setting is destined to be abandoned as a single agent, and its right place in HR+ MBC patients management needs to be urgently refined. Based on preclinical and early clinical trial results, its mechanism of action and pharmacokinetic properties make it an ideal backbone for combination therapies contributing to overcome or delaying endocrine resistance. Rational combinations with other therapies, such as *PI3K* inhibitors, HER2-directed therapies, and immunotherapy, are being explored. The emerging data also suggest a potential use of CDK4/6-targeted approaches in neoadjuvant settings (Supplementary Table S1). Different clinical trials are also ongoing to assess the safety and efficacy of CDK4/6 inhibitors alone or in combination with chemotherapy in different groups of patients. These trials, together with future comparative studies and biomarker analyses, are indispensable to better select patients who derive the greatest benefit from a specific class of CDK 4/6 inhibitors.

## Figures and Tables

**Table 1:** Main characteristics and outcomes of the seven eligible trials included for meta-analysis.

Study and First Author	Publication Year	Phase	Setting	Post-Menopausal	RR (%)	CB (%)	Median PFS (months)	Median OS (months)
PALOMA-2 Finn [12]	2016	III	First-line therapy for MBC in patients not treated before for their metastatic disease.	Yes	42.1 vs. 34.7	84.9 vs. 70.3	24.8 vs. 14.5	NR
MONALEESA-2 Hotobagyi [13]	2016 2018	III	First-line therapy for locally advanced and MBC. Patients who had not received previous systemic therapy for advanced disease were eligible. Previous neoadjuvant or adjuvant therapy with a nonsteroidal AI was not allowed unless the disease-free interval was more than 12 months	Yes	40.7 vs. 27.5 42.5 vs. 28.7	79.6 vs. 72.8 79.9 vs. 73.1	NR vs. 14.7 25.3 vs. 16	NR NR vs. 33
MONARCH-3 Goetz [14]	2017	III	First-line therapy for locally advanced and MBC (endocrine therapy in the neoadjuvant or adjuvant setting was permitted if the patient had a disease-free interval 12 months from the completion of endocrine therapy)	Yes	48.2 vs. 34.5	78 vs. 71.5	NR vs. 14.7	NR
MONALEESA-7 Tripathy [15]	2018 2019	III	First-line therapy for locally advanced or MBC (endocrine therapy and chemotherapy in the adjuvant or neoadjuvant setting was permitted, as was up to one line of chemotherapy for advanced disease).	Not (only pre and perimenopausal women were included and treated with goserelin)	41 vs. 30	79 vs. 70	23.8 vs. 13.0	NR NR vs. 40.9
FALCON Robertson [5]	2016	III	First-line therapy for locally advanced or MBC (no previous adjuvant therapy was admitted, only a first-line CHT for metastatic disease was accepted)	Yes	46 vs. 45	78 vs. 74	16.6 vs. 13.8	NR
SWOG Mehta [26]	2012 2019	III	First line for de novo MBC or recurrent MBC after 12 months by the end of adjuvant CHT or HT	Yes	27 vs. 22	73 vs. 70	15 vs. 13.5 15 vs. 13.5	47.7 vs. 41.3 49.8 vs. 42
FACT Bergh [27]	2012	III	First-line therapy in recurrent MBC after or during primary treatment (with or without HT, CHT, RT). Patients treated with an adjuvant AI had to be relapse-free for more than one year after completion of this type of endocrine therapy (30.2% of the patients in the experimental arm were endocrine naive)	Yes (only 3% of patients wer in the premenopausal status and were treated with GnRH agonists)	31.8 vs. 33.6	55 vs. 55.1	10.8 vs. 10.2	37.8 vs. 38.2

AI: aromatase inhibitor; CB: clinical benefit; CHT: chemotherapy; HT: hormonal therapy; MBC: metastatic breast cancer; GnRH, gonadotropin-releasing hormone; NR: not reported; OS: overall survival; PFS: progression-free survival; RR: response rate; RT: radiotherapy.

**Table 2:** Main adverse effects registered in each trial.

Study	Treatment	AEs	G3 (%)	G4 (%)
PALOMA-2	Palbociclib–letrozole	Any AEs	62.2%	13.5%
		Neutropenia	56.1%	10.4%
		Leukopenia	24.1%	0.7%
	Placebo–letrozole	Any AEs	22.1%	2.3%
		Neutropenia	0.9%	0.5%
MONALEESA-2	Ribociclib–letrozole	Neutropenia	52.4%	9.6%
		Abnormal LFT	8.4%	1.8%
		Leukopenia	20.1%	1.2%
	Placebo–letrozole	Abnormal LFT	2.4%	–
		Neutropenia, anemia, arthralgia	1.2%	–
MONARCH-3	Abemaciclib–nonsteroidal AI	Any AEs	51.7%	6.7%
		Neutropenia	22.0%	1.8%
		Leukopenia	8.3%	0.3%
		ALT increase	6.1%	0.3%
	Placebo–nonsteroidal AI	Any AEs	22.4%	2.5%
		Neutropenia	0.6%	0.6%
MONALEESA-7	Ribociclib group	Any AEs	63%	14%
		Neutropenia	51%	10%
		Leukopenia	13%	1%
	Placebo group	Any AEs	26%	4%
		Neutropenia	3%	1%
FALCON Robertson	Fulvestrant	Arthralgia (17%)		
		Hot flush, fatigue, nausea (11%)		
		Back pain (9%)		
	Anastrozole	Arthralgia, hot flush, nausea (10%)		
SWOG Mehta	Anastrozole	Musculoskeletal pain, fatigue, hot flashes, mood alterations, GI symptoms	15% (each 1–4%)	
	Anastrozole–fulvestrant	Musculoskeletal pain, fatigue, hot flashes, mood alterations, GI symptoms	13% (each 1–4%)	
FACT Bergh	Anastrozole	GI symptoms (25.2%)		
		Joint disorders (27.6%)		
		Hot flashes (13.8%)		
	Anastrozole–fulvestrant	GI symptoms (28.9%)		
		Joint disorders (26.6%)		
		Hot flashes (24.6%)		

ALT: alanine aminotransferase; AE: adverse event; AI: aromatase inhibitor; GI: gastrointestinal; G3: grade 3; G4: grade 4; LFT: liver function test.

**Table 3:** Main characteristics of the patients included in the randomized trials evaluated for the present network meta-analysis.

Characteristics	PALOMA-2, n (%)		MONALEESA-2, n (%)		MONARCH-3, n (%)		MONALEESA-7, n (%)		FALCON ^a^, n (%)		SWOG ^b^, n (%)		FACT4, n (%)	
	L. + Palb.	L.	L. + Rib.	L.	L or A. + Abem.	L. or A.	Rib. + T or nAIs	T or nAIs	F.	A.	F.+ A.	A.	F. + A.	A.
No. of patients	444	222	334	334	328	165	335	337	230	232	345	349	258	256
Age:														
• Median (range), years	62(3089)	61 (28–88)	62 (23–91)	63 (29–88)	63 (38–87)	63 (32–88)	43 (25–58)	45 (29–58)	64 (38–87)	62 (36–90)	65 (36–91)	65 (27–92)	65 (33–86)	63 (36–90)
• <65 years	263 (59.2)	141 (63.5)	NR	NR	NR	NR	NR	NR	NR	NR	NR	NR	124 (48.1)	145 (56.6)
• ≥65 years	181 (40.8)	81 (36.5)	NR	NR	NR	NR	NR	NR	108 (47)	91 (39)	NR	NR	134 (51.9)	111 (43.3)
Ethnicity:														
• White	344 (77.5)	172 (77.5)	269 (80.5)	280 (83.8)	186 (56.7)	102 (61.8)	187 (56)	201 (60)	175 (76)	174 (75)	NR	NR	242 (93.8)	237 (92.6)
• Asian	65 (14.6)	30 (13.5)	28 (8.4)	23 (6.9)	103 (31.4)	45 (27.3)	99 (30)	99 (29)	36 (16)	34 (15)	NR	NR	4 (1.6)	2 (0.8)
• Black	8 (1.8)	3 (1.4)	10 (3.0)	7 (2.1)	NR	NR	10 (3)	9 (3)	NR	NR	NR	NR	1 (0.4)	2 (0.8)
• Other	27 (6.1)	17 (7.7)	27 (8.1)	24 (7.2)	11 (3.4)	7 (4.2)	39 (12)	28 (8)	19 (8)	24 (10)	NR	NR	11 (4.3)	15 (5.9)
Hormone receptor status:														
• ER+, PgR+	NR	NR	NR	NR	255 (77.7)	127 (77.0)	290 (87)	288 (85)	175 (76)	179 (77)	NR	NR	193 (74.8)	195 (76.2)
• ER+, PgR−	NR	NR	NR	NR	70 (21.3)	36 (21.8)	NR	NR	44 (19)	43 (19)	NR	NR	60 (23.3)	51 (19.9)
• Unknown	NR	NR	NR	NR	NR	NR	NR	NR	10(4)	7 (3)	NR	NR	4 (1.6)	6 (2.3)
• ER−PgR+	NR	NR	NR	NR	NR	NR	NR	NR	1 (<1)	3 (1)	NR	NR	1 (0.4)	4 (1.6)
Performance status:														
• ECOG 0	257 (57.9)	102 (45.9)	205 (61.4)	202 (60.5)	192 (58.5)	104 (63.0)	245 (73)	255 (76)	117 (51)	115 (50)	NR	NR	NR	NR
• ECOG 1	178 (40.1)	117 (52.7)	129 (38.6)	132 (39.5)	136 (41.5)	61 (37.0)	87 (26)	78 (23)	106 (46)	105 (45)	NR	NR	NR	NR
• ECOG 2	9 (2.0)	3 (1.4)	NR	NR	NR	NR	0	1 (<1)	7 (3)	12 (5)	NR	NR	NR	NR
• ECOG > 2	NR	NR	NR	NR	NR	NR	NR	NR	NR	NR	NR	NR	NR	NR
• Unavailable	NR	NR	NR	NR	NR	NR	3 (1)	3 (1)	NR	NR	NR	NR	NR	NR
Disease stage:														
• I	51 (11.5)	30 (13.5)	NR	NR	NR	NR	NR	NR	NR	NR	NR	NR	NR	NR
• II	137 (30.9)	68 (30.6)	NR	NR	NR	NR	NR	NR	NR	NR	NR	NR	NR	NR
• III	72 (16.2)	39 (17.6)	1 (0.3)	3 (0.9)	NR	NR	NR	NR	NR	NR	NR	NR	NR	NR
• IV	138 (31.1)	72 (32.4)	333 (99.7)	331 (99.1)	NR	NR	NR	NR	202 (88)	200 (86)	NR	NR	245 (95)	242 (94.5)
• Unknown	36 (8.1)	1 (0.5)	NR	NR	NR	NR	NR	NR	NR	NR	NR	NR	NR	NR
Site of metastasis:														
• Bone only	103 (23.2)	48 (21.6)	69 (20.1)	78 (23.4)	70 (21.3)	39 (23.6)	81 (24)	78 (23)	24 (10)	24 (10)	76 (22.0)	75 (21.5)	63 (24.4)	71 (27.7)
• Visceral	214 (48.2)	110 (49.5)	197 (59.0)	196 (58.7)	172 (52.4)	89 (53.9)	193 (58)	188 (56)	135 (59)	119 (51)	167 (48.4)	181 (51.9)	134 (51.9)	124 (48.4)
• Non- visceral	230 (51.8)	112 (50.5)	NR	NR	NR	NR	NR	NR	60 (26)	81 (35)	102 (29.6)	93 (26.6)	195 (28.1)	NR
• Lymph nodes	NR	NR	133 (39.8)	123 (36.8)	NR	NR	142 (42)	158 (47)	NR	NR	NR	NR	NR	NR
• Other	NR	NR	45 (12.9)	33 (9.9)	86 (26.2)	37 (22.4)	8 (2)	8(2)	11 (4)	8 (4)	NR	NR	1 (0.4)	1 (0.4)
Measurable disease:														
• Yes	NR	NR	NR	NR	267 (81.4)	130 (78.8)	NR	NR	193 (84)	196 (84)	188 (54.5)	188 (53.9)	129 (50.0)	113 (44.1)
• No	NR	NR	NR	NR	61 (18.6)	35 (21.2)	NR	NR	NR	NR	157 (45.5)	161 (46.1)	129 (50.0)	143 (55.9)
Disease-free interval:														
• *De novo*	167 (37.6)	81 (36.5)	114 (34.1)	113 (33.8)	NR	NR	136 (41)	134 (40)	NR	NR	NR	NR	NR	NR
• ≤12 months	99 (22.3)	48 (21.6)	4 (1.2)	10 (3.0)	NR	NR	23 (7)	13 (4)	NR	NR	NR	NR	14 (5.4)	18 (7.0)
• >12 months	178 (40.1)	93 (41.9)	216 (64.7)	210 (62.9)	NR	NR	176 (53)	190 (56)	NR	NR	NR	NR	85 (32.9)	78 (30.5)
• Unknown	NR	NR	NR	1 (0.3)	NR	NR	NR	NR	NR	NR	NR	NR	NR	NR
No. of disease sites														
• 0	NR	NR	2 (0.6)	1 (0.3)	NR	NR	1 (<1)	NR	NR	NR	NR	NR	NR	NR
• 1	138 (31.1)	66 (29.7)	100 (29.9)	117 (35.0)	96 (29.3)	47 (28.5)	112 (33)	117 (35)	NR	NR	NR	NR	NR	NR
• 2	117 (26.4)	52 (23.4)	118 (35.3)	103 (30.8)	76 (23.2)	42 (25.5)	106 (32)	99 (29)	NR	NR	NR	NR	NR	NR
• 3	112 (25.2)	61 (27.5)	114 (34.1)	113 (33.8)	154 (47)^2^	75 (45.5)^2^	116 (35)	121 (36)	NR	NR	NR	NR	NR	NR
• ≥4	77 (17.3)	43 (19.4)	NR	NR	NR	NR	NR	NR	NR	NR	NR	NR	NR	NR
Prior chemotherapy:														
• Adjuvant	180 (40.5)	89 (40.1)	146 (43.7)	145 (43.4)	125 (38.1)	66 (40.0)	138 (41)	138 (41)	35 (15)	27 (12)	103 (29.9)	129 (37.0)	108 (41.9)	127 (49.6)
• Neoadjuvant	54 (12.2)	32 (14.4)	*	*	*	*	*	*	11 (5)	16 (7)	NR	NR	NR	NR
• Palliative	NR	NR	NR	NR	NR	NR	47 (14)	47 (14)	36 (16)	43 (19)	NR	NR	NR	NR
• None	NR	NR	NR	NR	203 (61.9)	99 (60.0)	150 (45)	152 (45)	NR	NR	242 (70.1)	220 (63.0)	NR	NR
Prior hormonal therapy:														
• Adjuvant	249 (56.1)	126 (56.8)	175 (52.4)	171 (51.2)	150 (45.7)	80 (48.5)	127 (38)	141 (42)	2 (1)	1 (<1)	139 (40.3)	141 (40.4)	180 (69.8)	168 (65.6)
• Neoadjuvant	NR		*	*										
• None	NR	NR	NR	NR	178 (54.3)	85 (51.5)	208 (62)	196 (58)	NR	NR	206 (59.7)	208 (59.6)	NR	NR
Type of adjuvant ET:														
• Tamoxifen	209 (47.1)	98 (44.1)	140 (41.9)	145 (43.4)	NR	NR	NR	NR	NR	NR	NR	NR	NR	NR
• Anastrozole	56 (12.6)	29 (13.1)	47 (14.1)	42 (12.6)	NR	NR	NR	NR	NR	NR	NR	NR	NR	NR
• Letrozole	36 (8.1)	16 (7.2)	34 (10.2)	25 (7.5)	NR	NR	NR	NR	NR	NR	NR	NR	NR	NR
• Exemestane	30 (6.8)	13 (5.9)	19 (5.7)	25 (7.5)	NR	NR	NR	NR	NR	NR	NR	NR	NR	NR
• Other	NR	NR	8 (2.4)	7 (2.1)	NR	NR	NR	NR	NR	NR	NR	NR	NR	NR

^a^ The experimental arm consisted of F 500 mg intramuscular injection; on days 0, 14, 28, then every 28 days thereafter. ^b^ The experimental arm consisted of F 500 mg on day 1 and 250 mg on days 14 and 28 and monthly thereafter. * Patients included among those of the adjuvant setting. L: letrozole; Palb.: palbociclib; Rib: Ribociclib; A: anastrozole; Abe: abemaciclib; T: tamoxifen; nAIs: non-steroidal aromatase inhibitors; F: fulvestrant; NR: not reported; ECOG: Eastern Cooperative Oncology Group; ER: estrogen receptor; PgR: progesteron receptor; +: positive.

## Supplementary Materials

The following are available online at https://www.mdpi.com/2072-6694/11/11/1661/s1, Table S1: The most important phase II or III ongoing clinical trials investigating CDK 4/6 inhibitors in breast cancer.
